# Berberine Alleviates *Citrobacter rodentium*-Induced Colitis with Reduced Epithelial Gasdermin E-Mediated Pyroptosis

**DOI:** 10.3390/ijms27156820

**Published:** 2026-07-29

**Authors:** Meiqi Zhang, Sixian Wei, Zheng Luo, Qiqi Li, Lingdong Kong, Shiqi Wu, Meiqi Jia, Yingying Wang, Yao Wang, Anlong Xu, Xin Jia

**Affiliations:** 1 School of Life Sciences, Beijing University of Chinese Medicine, Beijing 100029, China; 2 School of Chinese Materia Medica, Beijing University of Chinese Medicine, Beijing 100029, China; 3 School of Chinese Medicine, Beijing University of Chinese Medicine, Beijing 100029, China; 4 Beijing Research Institute of Chinese Medicine, Beijing University of Chinese Medicine, Beijing 100029, China; 5 Institute of Advanced Studies Hong Kong, Sun Yat-sen University, Hong Kong 999077, China

**Keywords:** Berberine, inflammatory bowel disease, GSDME, pyroptosis

## Abstract

Inflammatory bowel disease (IBD) is a chronic inflammatory condition of the intestine. Although current therapies can provide symptomatic relief, they are often associated with high costs and adverse effects. *Coptis chinensis* Franch., a traditional Chinese medicine long used for inflammatory intestinal conditions such as bacterial dysentery, is a promising alternative. However, the specific alkaloid components that inhibit cell death and alleviate colitis remain unclear. Major isoquinoline alkaloids from *Coptis chinensis* Franch. were screened in a TNF-α/cycloheximide (CHX)-induced epithelial cell death model using CCK-8 and Annexin V staining. Caspase-3 and GSDME cleavage, HMGB1 release, phosphorylation of ERK and JNK, and inflammatory gene expression were assessed by Western blotting and qPCR. In vivo, a *Citrobacter rodentium*-induced colitis model was established and mice were treated with a specific alkaloid. Disease severity, histopathological injury, bacterial burden, barrier-related structural markers, and colonic GSDME cleavage were then evaluated. Berberine (BBR) exhibited the strongest cytoprotective activity in the screening system, reducing epithelial cell death, reversing pyroptosis-associated morphology, and inhibiting GSDME cleavage. BBR also reduced HMGB1 release, attenuated the phosphorylation of ERK and JNK, and downregulated pro-inflammatory gene expression. In the *C. rodentium*-induced infectious colitis model, BBR alleviated body weight loss, diarrhea, colon shortening, and histopathological injury without significantly reducing bacterial burden under the present experimental conditions. In parallel, BBR enhanced *Tjp1* and *Muc2* expression, and diminished colonic GSDME cleavage in vivo. Collectively, these findings suggest that BBR is a promising candidate for colitis involving bacterial infection-associated epithelial pyroptosis, potentially via GSDME modulation.

## 1. Introduction

Inflammatory bowel disease (IBD), including ulcerative colitis and Crohn’s disease, is a chronic relapsing inflammatory disorder of the gastrointestinal tract [1,2,3]. In addition to genetic susceptibility, dysbiosis, immune dysregulation, and environmental factors, disruption of the intestinal epithelial barrier is recognized as a central event that amplifies mucosal inflammation and disease progression [4,5]. Intestinal epithelial cells (IECs) form both the physical and chemical barriers that maintain gut homeostasis, and excessive epithelial injury promotes luminal antigen exposure, inflammatory cell recruitment, and persistent tissue damage [6,7,8]. Therefore, preserving epithelial integrity is an important therapeutic strategy in both IBD and other forms of colitis characterized by barrier disruption.

Pyroptosis is a lytic form of programmed cell death mediated by gasdermin family proteins [5,9,10]. Among them, gasdermin E (GSDME) can be cleaved by Caspase-3 to release its pore-forming N-terminal fragment, thereby converting apoptotic signals into pyroptotic outcomes [11,12,13]. In the canonical inflammasome pathway, activation of the NOD-like receptor pyrin domain-containing 3 (NLRP3) inflammasome recruits and activates Caspase-1, which subsequently cleaves gasdermin D (GSDMD) to induce pyroptosis and promotes the maturation of IL-1β and IL-18 [14]. Unlike GSDMD, which is widely expressed in immune cells, GSDME is preferentially expressed in intestinal epithelial cells. Increasing evidence indicates that GSDME-mediated epithelial pyroptosis contributes to intestinal inflammation by compromising barrier integrity and promoting the release of pro-inflammatory mediators such as high mobility group box 1 (HMGB1), which further activates the downstream extracellular signal-regulated kinase (ERK) and c-Jun N-terminal kinase (JNK) pathway [15,16,17]. Accordingly, GSDME-related epithelial injury has emerged as a potentially relevant mechanism in colitis [18,19,20].

*Coptis chinensis* Franch. is a well-known traditional Chinese medicine that has long been used for intestinal disorders such as dysentery and diarrhea [21,22,23]. In traditional practice, it is prescribed either as a single herb or as part of classic formulas for damp-heat-related gastrointestinal diseases [24,25,26,27]. Modern pharmacological studies have shown that the major constituents of *Coptis chinensis* Franch. are isoquinoline alkaloids, including Berberine (BBR), Coptisine, Epiberberine, Palmatine, Jatrorrhizine, and Magnoflorine, many of which exhibit anti-inflammatory and barrier-protective effects in experimental colitis [28,29,30,31,32,33,34,35]. However, it remains unclear which major alkaloid shows the strongest protective activity in a cell death-based screening setting and whether such protection is associated with suppression of GSDME-related epithelial pyroptosis.

In the present study, we screened major isoquinoline alkaloids from *Coptis chinensis* Franch. using a TNF-α/cycloheximide (CHX)-induced colonic epithelial cell death model and identified BBR as the most active compound under our experimental conditions. We then evaluated its activity in a *Citrobacter rodentium*-induced colitis model, an infection-driven setting characterized by epithelial injury and inflammatory amplification. Here, we show that BBR alleviated colitic injury, reduced epithelial GSDME cleavage, restrained HMGB1/ERK/JNK inflammatory signaling, and improved barrier-related markers without significantly reducing bacterial burden under the present experimental conditions. These findings suggest that BBR protects against epithelial injury in infection-associated colitis, potentially via modulation of the GSDME-related pathway.

## 2. Results

### 2.1. Identification of Berberine as the Lead Alkaloid Against Epithelial Cell Death

**Figure 1:**
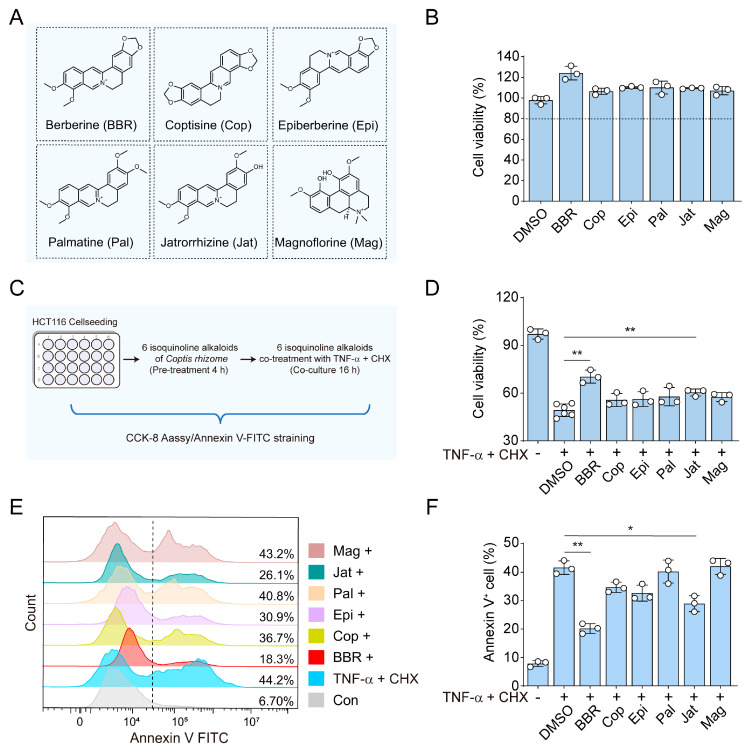
The effects of six isoquinoline alkaloids in *Coptis chinensis* Franch. on cell death. (**A**) Chemical structures of the six isoquinoline alkaloids. (**B**) Assessment of cytotoxic effects of the six isoquinoline alkaloids (at 5 μM) on HCT116 cells following 24 h of exposure, determined by the CCK-8 assay (*n* = 3). (**C**) Schematic diagram of the experimental procedure. (**D**) Cell viability analysis of HCT116 cells pre-incubated with each of the six alkaloids (5 μM) for 4 h, followed by combined treatment with TNF-α/CHX for 16 h. Cell viability was quantified using the CCK-8 method (*n* = 3 or 6). (**E**,**F**) Cell death analysis of HCT116 cells pre-incubated with each of the six alkaloids (5 μM) for 4 h, followed by combined treatment with TNF-α/CHX for 16 h. Cell death was measured by flow cytometry using Annexin V-FITC staining (*n* = 3). Data are presented as mean ± SD; * *p* < 0.05, and ** *p* < 0.01 versus model group.

To identify the most active alkaloid from *Coptis chinensis* Franch. against epithelial cell death, six major isoquinoline alkaloids, including BBR, Coptisine (Cop), Epiberberine (Epi), Palmatine (Pal), Jatrorrhizine (Jat), and Magnoflorine (Mag), were selected for screening. Their chemical structures are shown in Figure 1A. First, the cytotoxicity of each compound was evaluated in HCT116 cells at a concentration of 5 μM using the CCK-8 assay. The results showed that after 24 h of treatment, cell viability remained above 95% for all alkaloids, indicating no significant cytotoxicity at this concentration (Figure 1B).

Subsequently, a TNF-α/CHX-induced colonic epithelial cell death model was employed to evaluate the inhibitory effects of various alkaloids from *Coptis chinensis* Franch. on cell death (Figure 1C). CCK-8 assay results showed that cell viability in the model group decreased to 46.4%, whereas treatment with BBR and Jat significantly restored viability to 70.0% and 61.6%, respectively, while the other alkaloids exhibited no significant protective effects (Figure 1D). These findings were further corroborated by Annexin V staining. Flow cytometry analysis revealed that the proportion of Annexin V-positive cells in the model group was approximately 44.2%, and treatment with BBR and Jat reduced this proportion to 18.3% and 26.1%, respectively (Figure 1E,F). Together, these data identified BBR as the lead alkaloid in the screening system and supported its selection for subsequent mechanistic and in vivo studies.

### 2.2. Berberine Reduces Pyroptosis-Associated Epithelial Cell Death and GSDME Cleavage

**Figure 2:**
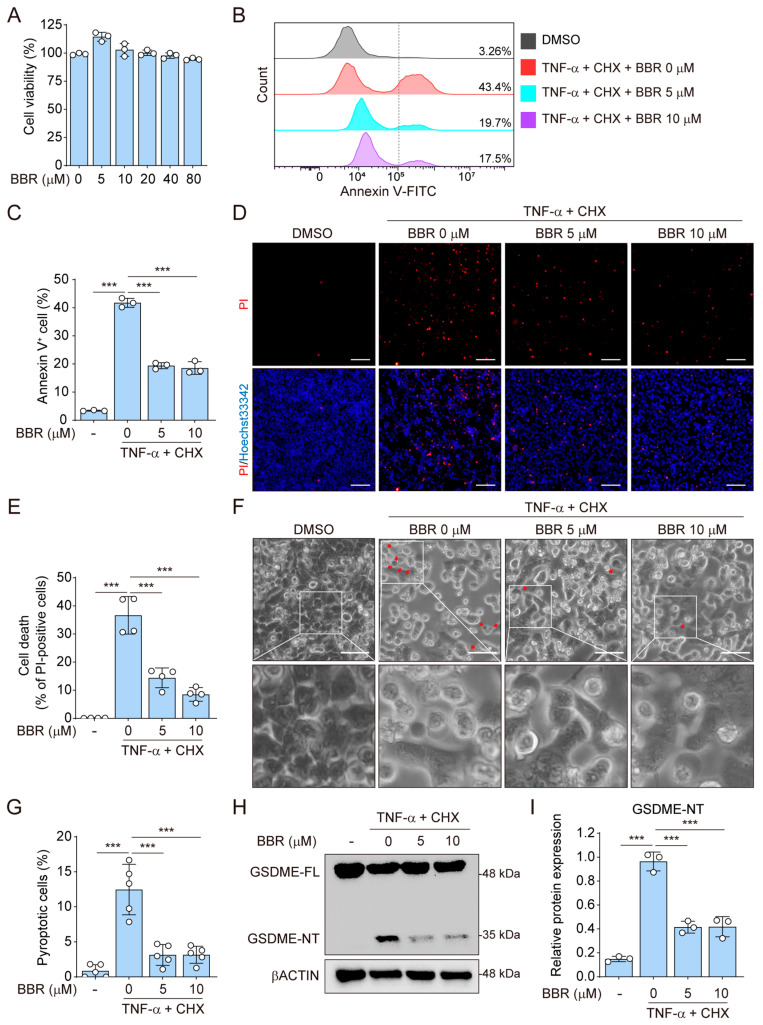
Berberine reduces pyroptosis-associated epithelial cell death and GSDME cleavage. (**A**) HCT116 cells were treated with BBR for 24 h and cell viability was measured by CCK-8 (*n* = 3). For (**B**–**I**), HCT116 cells were pretreated with BBR for 4 h and subsequently co-incubated with TNF-α/CHX for 16 h. (**B**–**E**) Cell death was evaluated via flow cytometry using Annexin V-FITC staining (**B**,**C**; *n* = 3) and by PI staining (**D**,**E**; scale bar 100 µm; *n* = 4). (**F**,**G**) Representative bright-field images show pyroptotic morphology (scale bar = 50 µm; *n* = 5). (**H**,**I**) Protein expression levels of full-length GSDME (GSDME-FL) and its N-terminal cleavage (GSDME-NT) were determined by Western blotting. The relative grayscale value of the GSDME-NT protein band was measured with ImageJ software (*n* = 3). Data are presented as mean ± SD. *** *p* < 0.001 versus model group.

Given the superior cytoprotective activity of BBR, subsequent experiments focused on this compound. HCT116 cells were exposed to graded concentrations of BBR over 24 h, after which cell viability was evaluated via the CCK-8 assay. Figure 2A indicates that BBR at doses of 0, 5, 10, 20, 40, and 80 μM did not significantly alter HCT116 cell viability. Two concentrations of BBR (5 and 10 μM), which showed no obvious cytotoxicity, were selected for further experiments. In the TNF-α/CHX-induced cell death model, flow cytometry analysis with Annexin V-FITC staining revealed that the percentage of Annexin V-positive cells in the model group was approximately 43.4%, whereas treatment with 5 µM and 10 µM BBR significantly reduced this proportion to 19.7% and 17.5%, respectively (Figure 2B,C). Propidium iodide (PI) staining further demonstrated a significant increase in PI-positive cells following TNF-α/CHX stimulation, which was attenuated by BBR treatment (Figure 2D,E). These results suggest that BBR exerts a protective effect against cell death. In HCT116 cells challenged with TNF-α/CHX, typical morphological signs of pyroptosis are evident, such as cellular swelling, and the formation of rupturing balloon-like membranes that collapse into a shrunken remnant (Figure 2F,G). In line with the decreased cell death, these morphological changes are reversed by BBR treatment (Figure 2F,G).

To further determine whether the observed protective effect involves the canonical pyroptosis pathway, we examined the cleavage of Caspase-1 and GSDMD by Western blotting. As shown in Figure S1, TNF-α/CHX stimulation and BBR treatment did not appreciably affect Caspase-1 or GSDMD cleavage, suggesting that BBR does not interfere with the canonical inflammasome-driven pyroptotic axis in this model. In contrast to the lack of effect on the canonical pathway, GSDME cleavage was markedly elevated in the model group, whereas BBR treatment suppressed this increase (Figure 2H,I). Importantly, this inhibitory effect of BBR on GSDME cleavage was further confirmed in the normal human colonic epithelial cell line NCM460 (Figure S2A,B), indicating that the suppressive action of BBR on GSDME is not restricted to HCT116 cells. Collectively, these findings indicate that BBR reduces epithelial pyroptosis in the TNF-α/CHX model, accompanied by reduced cleavage of GSDME.

### 2.3. Berberine Suppresses Downstream HMGB1/ERK/JNK Inflammatory Signaling

**Figure 3:**
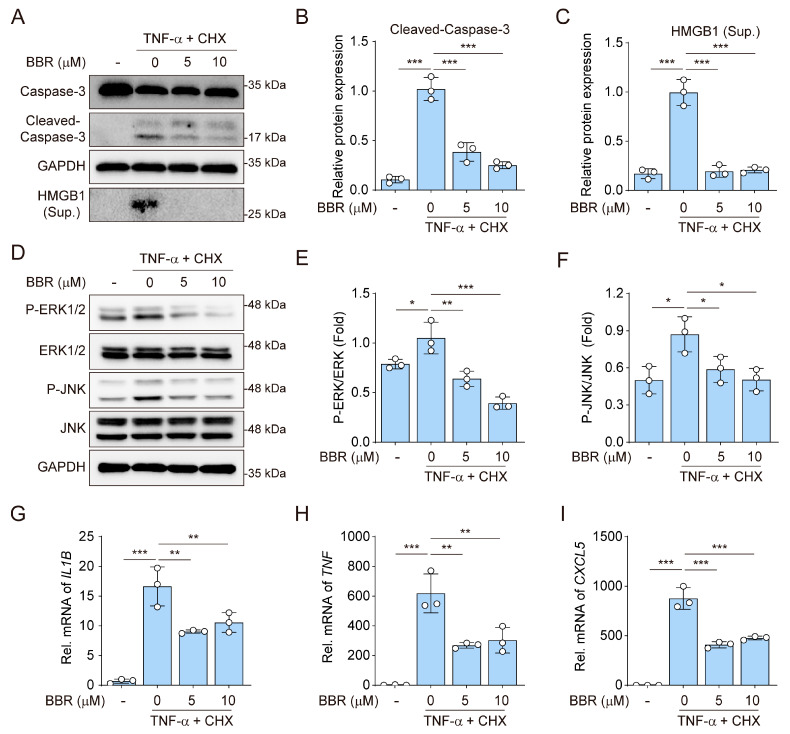
Berberine suppresses downstream HMGB1/ERK/JNK inflammatory signaling. (**A**–**C**) Western blotting was performed to detect the protein levels of Caspase-3, its cleaved form (cleaved Caspase-3), and HMGB1 in both HCT116 cell lysates and culture supernatants. The relative grayscale value of the cleaved Caspase-3 and HMGB1 protein bands was measured with ImageJ software (*n* = 3). (**D**–**F**) Western blotting was performed to detect the protein levels of P-ERK1/2, ERK1/2, P-JNK, and JNK in HCT116 cell lysates. The relative grayscale value of the P-ERK1/2 and P-JNK protein bands was measured with ImageJ software (*n* = 3). (**G**–**I**) Transcript levels of the inflammatory mediators *IL1B*, *TNF*, and *CXCL5* were examined via qPCR (*n* = 3). Data are presented as mean ± SD. * *p* < 0.05, ** *p* < 0.01, and *** *p* < 0.001 versus model group.

Based on previous reports, GSDME cleavage by Caspase-3 leads to HMGB1 release, which in turn facilitates colitis progression [11,15]. Accordingly, we assessed active Caspase-3 in cell lysates and HMGB1 release in cell supernatant. Western blotting revealed elevated levels of cleaved Caspase-3 and HMGB1 in the model group, whereas BBR treatment significantly reduced the cleavage of Caspase-3 and the release of HMGB1 (Figure 3A–C). Extracellular HMGB1 has been reported to trigger ERK and JNK signaling cascades via its receptors, thereby driving colonic mucosal inflammation progression [15,36,37]. To further explore the downstream inflammatory signaling underlying the protective action of BBR, we examined ERK and JNK phosphorylation along with inflammatory gene expression in the TNF-α/CHX model. Western blotting showed that BBR treatment markedly attenuated the levels of phosphorylated ERK1/2 (P-ERK1/2) and P-JNK (Figure 3D–F). Subsequently, qPCR was conducted to assess the impact of BBR on inflammatory cytokine expression. The findings indicated that, relative to the model group, BBR remarkably downregulated the mRNA levels of *IL1B*, *TNF*, and *CXCL5* (Figure 3G–I). Together, these data place BBR-associated protection within a downstream HMGB1/ERK/JNK-driven inflammatory signaling cascade.

### 2.4. Berberine Alleviates Citrobacter rodentium-Induced Colitic Injury In Vivo

**Figure 4:**
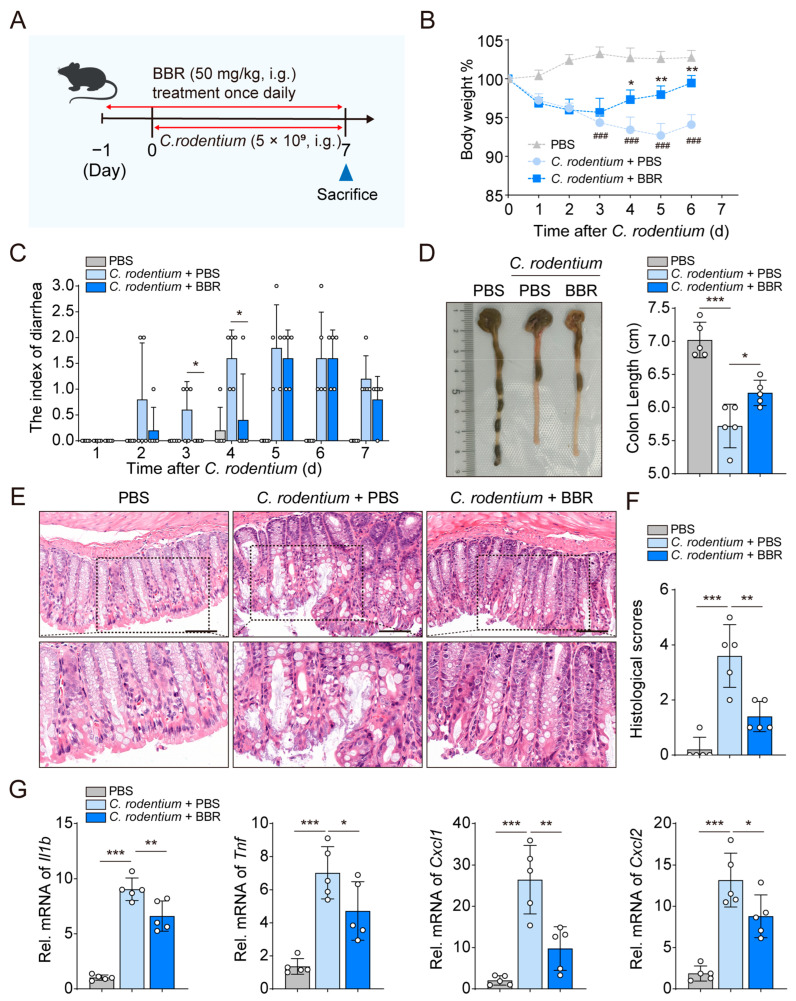
Berberine alleviates *C. rodentium*-induced colitic injury in vivo. (**A**) Schematic diagram illustrating the in vivo experimental timeline and treatment regimen for animal studies. (**B**) Body weight change (*n* = 5). (**C**) Diarrhea index (*n* = 5). (**D**) Colon length at day 7 (*n* = 5). (**E**,**F**) Histological examination of colonic sections with H&E staining (scale bar = 100 µm; *n* = 5). (**G**) Expression of inflammatory factors (*Il1b*, *Tnf*, *Cxcl1*, and *Cxcl2*) in colon tissues was measured by qPCR (*n* = 5). Data are presented as mean ± SD. ^###^ *p* < 0.001 versus control group; * *p* < 0.05, ** *p* < 0.01, and *** *p* < 0.001 versus model group.

To determine whether BBR protects against infection-associated colitic injury in vivo, we established a *C. rodentium*-induced colitis model and treated mice with BBR daily (Figure 4A). *C. rodentium* is an attaching and effacing enteric pathogen that specifically colonizes the colonic epithelium, inducing crypt hyperplasia, immune cell infiltration, diarrhea, and other pathological features, and thus imitates aspects of enteropathogenic *Escherichia coli* infection and IBD [38]. BBR treatment did not significantly affect food consumption or water intake during the experimental period (Figure S3A,B). In comparison with uninfected control mice, those challenged with *C. rodentium* displayed a marked reduction in body weight alongside an elevated diarrhea score (Figure 4B,C). Additionally, the colonic length of infected mice was significantly shorter than that of the uninfected control group (Figure 4D). Importantly, we observed that the pathological changes induced by *C. rodentium* were notably ameliorated following BBR administration (Figure 4B–D). Moreover, histopathological examination of colon tissue further demonstrated that *C. rodentium* infection resulted in severe structural abnormalities, such as epithelial disruption, crypt loss, a pronounced decrease in goblet cell numbers, and inflammatory cell infiltration (Figure 4E,F). All of these tissue injuries were conspicuously alleviated in mice receiving BBR treatment (Figure 4E,F).

Beyond the evaluation of colonic epithelial damage, the regulatory effect of BBR on inflammatory mediators was also investigated. At 7 days post-infection, colonic transcript levels of *Il1b*, *Tnf*, *Cxcl1*, and *Cxcl2* were substantially higher in *C. rodentium*-infected mice relative to uninfected controls (Figure 4G). However, BBR treatment effectively suppressed the expression of these pro-inflammatory cytokines and chemokines (Figure 4G). These findings indicate that BBR mitigates infection-associated colitic injury and inflammatory responses in vivo.

### 2.5. Berberine Exerts Protection Independently of Bacterial Burden Reduction

**Figure 5:**
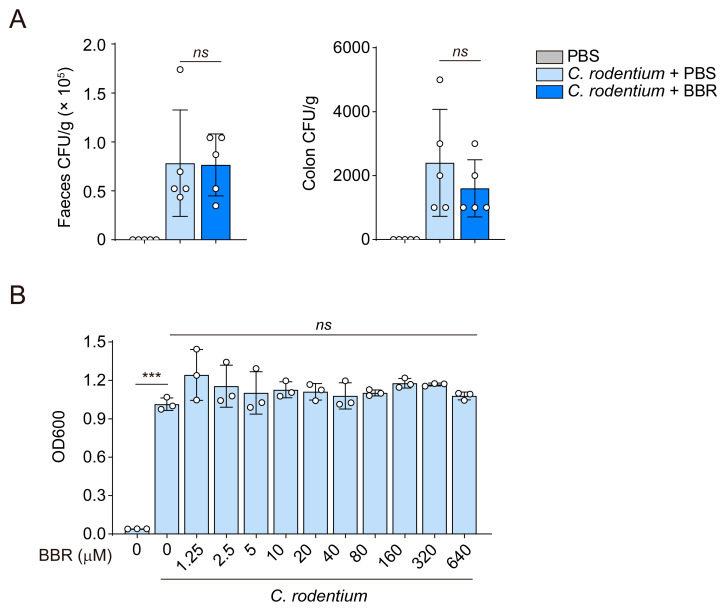
The impact of Berberine on pathogen clearance. (**A**) Quantification of CFU per gram of feces and colon (*n* = 5). (**B**) OD600 measurement was taken following a 24 h incubation of *C. rodentium* with BBR in vitro (*n* = 3). Data are presented as mean ± SD. *ns* = not significant; *** *p* < 0.001 versus model group.

To determine whether the protective action of BBR correlated with changes in bacterial load, we measured *C. rodentium* loads in infected mice and assessed bacterial growth in vitro. On day 7 post-infection, BBR treatment did not markedly reduce fecal *C. rodentium* burden (Figure 5A). Consistently, the bacterial counts within colonic tissue did not change substantially in BBR-treated mice (Figure 5B). Meanwhile, in vitro antibacterial experiments revealed that the presence of BBR had no effect on *C. rodentium* growth. Together, these data indicate that the beneficial effect of BBR in this model cannot be primarily attributed to a pronounced decrease in bacterial burden.

### 2.6. Berberine Improves Barrier-Associated Structural Markers and Reduces Colonic GSDME Cleavage In Vivo

**Figure 6:**
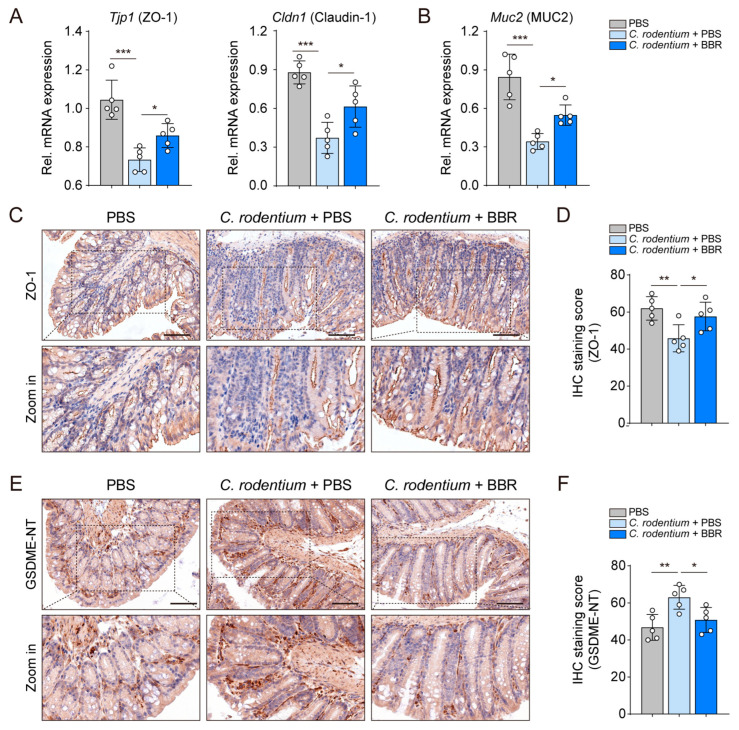
Berberine improves barrier-associated structural markers and reduces colonic GSDME cleavage in vivo. (**A**,**B**) *Zo-1*, *Cldn1*, and *Muc2* mRNA were measured by qPCR (*n* = 5). (**C**,**D**) Representative IHC images and quantification of positive area of ZO-1 in colon tissues (*n* = 5). (**E**,**F**) Representative IHC images and quantification of positive area of GSDME-NT in colon tissues (*n* = 5). Scale bar = 100 µm. Data are presented as mean ± SD. * *p* < 0.05, ** *p* < 0.01, and *** *p* < 0.001 versus model group.

To further assess whether BBR influences epithelial barrier integrity, we measured tight junction- and mucus-related markers in colonic tissues from infected mice. Using qPCR, we found that colitis induced by *C. rodentium* infection caused a pronounced decrease in the transcript levels of *Tjp1* (ZO-1) and *Cldn1* (claudin-1) in colonic tissue samples (Figure 6A). Notably, treatment with BBR effectively counteracted this downregulation (Figure 6A). MUC2, secreted by goblet cells, is the primary component of the intestinal mucus layer and serves as the first chemical barrier that separates luminal microorganisms from the intestinal epithelium. Deficiency of MUC2 directly results in direct contact between commensal bacteria and the epithelial surface [39,40]. qPCR analysis indicated that *Muc2* mRNA expression was markedly decreased in colonic tissues following *C. rodentium* infection, whereas treatment with BBR partially restored *Muc2* expression (Figure 6B). IHC staining confirmed these findings. Colon sections from mice with *C. rodentium*-induced colitis exhibited substantially diminished immunoreactivity for ZO-1, whereas the BBR-treated group showed continuous and stronger staining patterns (Figure 6C,D). Additional IHC analysis demonstrated that *C. rodentium* infection triggered excessive cleavage of GSDME, which was potently suppressed by BBR treatment (Figure 6E,F). Taken together, these findings indicate that BBR treatment upregulated the expression of barrier-associated structural markers while simultaneously reducing colonic GSDME cleavage in the infection model.

## 3. Discussion

The present study identifies BBR as the most active alkaloid in a screening system based on colonic epithelial cell death among major isoquinoline alkaloids from *Coptis chinensis* Franch. More importantly, our findings link the protective effect of BBR in *C. rodentium*-induced colitis to reduced GSDME-related epithelial injury, thereby highlighting a potential mechanism through which this traditional medicinal herb may alleviate infection-associated colitic damage.

BBR has been widely reported to exert protective effects in dextran sodium sulfate (DSS)-induced experimental colitis through multiple mechanisms, including modulation of gut microbiota, suppression of inflammatory signaling, and preservation of epithelial barrier function [28,29,30,31,41,42,43,44,45]. Our study extends the action of BBR from conventional DSS-based settings to an infection-driven *C. rodentium*-induced colitis model, which may better reflect epithelial injury and inflammatory amplification relevant to dysentery-like intestinal disorders traditionally treated with *Coptis chinensis* Franch.

An important observation in this study is that BBR improved body weight loss, diarrhea, histopathological injury, inflammatory cytokine expression, and barrier-related markers without significantly reducing bacterial burden under the experimental conditions tested. This result suggests that the protective effect of BBR in this model is more likely to reflect modulation of host injury responses than direct antibacterial activity. This burden-independent protection suggests a host-directed mode of action in which limiting epithelial pyroptotic damage and barrier disruption may be more important than directly suppressing bacterial growth. Therefore, limiting epithelial pyroptosis-associated barrier disruption may represent an important component of the beneficial activity of BBR.

GSDME-mediated pyroptosis has recently attracted increasing attention in intestinal disease [15,16,19,46,47]. Previous studies have shown that GSDME is upregulated or excessively activated in colitis-related settings and contributes to epithelial damage, HMGB1 release, and inflammatory amplification [15]. Consistent with this literature, our data show that BBR reduces GSDME cleavage in vitro and in vivo, together with suppression of HMGB1 release and downstream ERK/JNK signaling. Although the inhibitory effect of BBR on GSDME cleavage has been confirmed, and BBR shows no appreciable effect on canonical pyroptosis mediated by Caspase-1 and GSDMD, the potential contribution of other cell death modalities, including apoptosis and necroptosis, has not been systematically ruled out. Further studies are warranted to clarify these possibilities. In addition, while our findings demonstrate a correlation between BBR treatment and reduced GSDME-mediated pyroptosis in the context of *C. rodentium* infection, the causal dependency of BBR’s protective effects on GSDME has not been established. Future investigations employing GSDME knockout mice or selective pharmacological inhibitors are required to definitively address this question.

However, our study has several limitations. HCT116 is a colorectal cancer cell line whose signaling pathways may diverge from those of normal intestinal epithelium. Although we have verified the inhibitory effect of BBR on GSDME cleavage in normal colonic epithelial cells, our in vitro findings still warrant further validation in more physiologically relevant primary models, such as colonic organoid. Furthermore, although the expression of barrier-associated structural markers was upregulated following BBR treatment, this did not translate into a demonstrable improvement in functional barrier integrity in vivo. Future studies employing FITC-dextran permeability assays are required to confirm the restoration of epithelial barrier integrity. Additionally, the upstream mechanism by which BBR limits GSDME cleavage remains unknown. Based on the previous literature, we hypothesize that AMP-activated protein kinase (AMPK) may be a key candidate, as BBR is a well-recognized AMPK activator and AMPK activation has been reported to significantly inhibit GSDME-mediated pyroptosis [48,49,50,51]. AMPK signaling was not directly examined in the present study, and this potential mechanism warrants further investigation.

## 4. Materials and Methods

### 4.1. Cell Culture

The HCT116 cell line (ATCC, Manassas, VA, USA) and NCM460 cell line (ATCC) were cultured in RPMI-1640 medium supplemented with 10% fetal bovine serum (FBS) and 1% penicillin–streptomycin (Gibco). Incubation was performed at 37 °C in a humidified atmosphere of 5% CO_2_. Culture medium, FBS, and cell culture additives were purchased from Gibco (Waltham, MA, USA).

### 4.2. Cell Stimulation

Berberine (PU0269-0025, Push Bio-Technology, Chengdu, China), Coptisine (PU0836-0025, Push Bio-Technology), Epiberberine (PU0861-0020, Push Bio-Technology), Palmatine (PU0916-0025, Push Bio-Technology), and Jatrorrhizine (PU0070-0025, Push Bio-Technology) were used in their hydrochloride salt forms, and Magnoflorine (PS1769-0025, Push Bio-Technology) was used in its iodide salt form; all are the most common stable preparations. HCT116 cells were pretreated with 5 μM isoquinoline alkaloids for 4 h and then co-treated with 20 ng/mL of TNF-α (AF-300-01A, PeproTech, Cranbury, NJ, USA) and 10 μg/mL of CHX (HY-12320, MCE, Washington, DC, USA) for 16 h to induce cell death.

### 4.3. Cell Viability Assay

Cell viability was assessed using the cell counting kit-8 (CCK-8). In brief, HCT116 cells were plated into 96-well plates at a density of 1 × 10^4^ cells per well and incubated overnight to allow attachment. The cells were then further treated with various alkaloids for 24 h. After treatment, 10 μL CCK-8 solution (CK04, DOJINDO, Kumamoto, Japan) was added per well, followed by incubation at 37 °C for 15–30 min. The absorbance at 450 nm was recorded with a microplate reader (Thermo Scientific, Waltham, MA, USA).

### 4.4. Flow Cytometry

Cell death was detected employing the Annexin V FITC Detection Kit (C1062, Beyotime, Shanghai, China). HCT116 cells were harvested and rinsed twice with PBS. Subsequently, 500 μL binding buffer suspension was added to the treated cells. Following this, 5 μL Annexin V-FITC was introduced into each group, followed by incubation of the cultures at room temperature for 5–15 min in the dark. Samples were then analyzed using a CytoFLEX flow cytometer (Beckman Coulter, Brea, CA, USA).

### 4.5. PI Staining

To examine the inflammatory cell death, HCT116 cells were stained for 10 min with PI (2 μg/mL, P4170, Sigma-Aldrich, Taufkirchen, Germany) and Hoechst33342 (5 μg/mL, A3472, APExBio Technology, TX, USA). Images of pyroptotic cells were acquired using a fluorescent microscope (DP 74, Olympuss, Tokyo, Japan).

### 4.6. Western Blotting

Western blotting was conducted following a previously described method [52]. Protein samples were separated on 12.5% SDS-polyacrylamide gels and then electro-transferred onto nitrocellulose membranes (PALL, New York, NY, USA). After blocking with 5% BSA for 1 h, the membranes were probed overnight at 4 °C with primary antibodies diluted in blocking buffer, followed by incubation with secondary antibodies for 1 h at room temperature. Protein signals were detected using the Immobilon ECL kit (Millipore, Waltham, MA, USA) and visualized on the Tanon-5200 Imaging System (Tanon, Shanghai, China). Furthermore, the band intensities were quantified using ImageJ software v1.54 (National Institutes of Health, Bethesda, MD, USA).

The antibodies used in the study are listed as follows: DFNA5/GSDME (ab215191, Abcam, Cambridge, UK); Caspase-3 (ab184787, Abcam); cleaved Caspase-3 (9664S, Cell Signaling Technology, Beverly, CA, USA); Beta Actin (66009-1-Ig, Proteintech, Chicago, IL, USA); Caspase-1 (3866S, Cell Signaling Technology); GSDMD (ab219800, Abcam); cleaved N-terminal GSDMD (ab215203, Abcam); GAPDH (60004-1-Ig, Proteintech); P-ERK1/2 (1202/Y204) (4370S, Cell Signaling Technology); ERK1/2 (ZH5003, Zhonghe, Beijing, China); P-JNK(T183/Y185) (4668S, Cell Signaling Technology); JNK (ZN5037, Zhonghe); HMGB1 (3935S, Cell Signaling Technology); and HRP-conjugated Goat Anti-Rabbit IgG (C650202, Sangon Biotech, Shanghai, China).

### 4.7. RNA Isolation and qPCR

Total RNA was isolated using TRIzol (Invitrogen, Waltham, MA, USA) according to a previously described method [52]. Complementary DNA was synthesized, and qPCR amplification was performed using the Evo M-MLV One Step RT-qPCR Mix (AG11763, Accurate Biology, Changsha, China) with gene-specific primers on a CFX96 Real-Time PCR Detection System (Bio-Rad, Carlsbad, CA, USA). The primer sequences employed are listed in Supplementary Table S1.

### 4.8. Mice

Male C57BL/6J mice (6–8 weeks old) were used in all assays. All animals were maintained in accordance with the ethical policies and procedures approved by the Beijing University of Chinese Medicine Institutional Animal Care and Use Committee (Ethics No. BUCM-2024061801-2181).

### 4.9. Generation of Citrobacter rodentium-Induced Colitis Model and BBR Treatment

*C. rodentium* (51459, ATCC) infection was induced in the mice as previously described. The mice were randomly allocated into three groups (*n* = 5 per group): normal control, *C. rodentium*-induced colitis model, and BBR-treated group (50 mg/kg). The mouse model of colitis was illustrated in Figure 4A. BBR was administered by gavage at 100 μL/day once daily, starting one day prior to *C. rodentium* challenge. On the following day, both the model and BBR-treated groups were orally gavaged with 5 × 10^9^ colony-forming units (CFU) of the pathogen. Body weight and diarrhea score were monitored daily until the study endpoint. Subsequently, the animals were sacrificed, and colonic length was measured. Colon specimens were rinsed in PBS and stored in appropriate solutions for subsequent analysis.

### 4.10. The Diarrhea Score

Fecal specimens were scored in a blinded manner on a scale from 1 to 4 (diarrhea index, DI) based on color, texture, and quantity, using the following criteria: DI = 1, normal; DI = 2, loose yellow-green; DI = 3, completely loose yellow-green; DI = 4, high amount of watery feces.

### 4.11. C. rodentium Determination in Mouse Feces and Tissues

For the determination of CFU, feces and colon were harvested, weighed, and homogenized in sterile PBS 7 days after *C. rodentium* infection. Homogenates were then plated on MacConkey agar (HB6238, Hopebio, Qingdao, China) for 24 h at 37 °C and *C. rodentium* colonies were counted for CFU determination by normalizing for volume and weight of the starting tissue.

### 4.12. Hematoxylin and Eosin (H&E) Staining

For H&E staining, mouse distal colon tissues were fixed in 4% paraformaldehyde. After fixation, specimens were rinsed under running tap water for 30 min to remove the fixative, followed by dehydration through a graded ethanol series. The dehydrated tissues were cleared in xylene and then embedded in paraffin blocks. Sections were cut at 5 μm thickness, deparaffinized in xylene, and rehydrated through a descending ethanol series. Slides were subsequently stained with hematoxylin and eosin and then dehydrated, cleared, and mounted with a coverslip. Images were captured using a super-resolution automatic tissue scanning microscope to assess crypt damage and inflammatory cell infiltration. Detailed scoring criteria are provided in Supplementary Table S2 [53].

### 4.13. Immunohistochemistry (IHC)

For IHC staining, paraffin-embedded mouse colon sections were deparaffinized and subjected to antigen retrieval. Endogenous peroxidase activity was quenched by treatment with hydrogen peroxide. Non-specific binding was blocked with 5% BSA. Thereafter, the sections were then incubated overnight at 4 °C with primary antibodies targeting GSDME N-terminal (ab222407, Abcam) and ZO-1 (21773-1-AP, Proteintech), followed by appropriate HRP-conjugated secondary antibodies, and positive immunoreactivity was revealed using a diaminobenzidine substrate. Finally, the sections were counterstained with hematoxylin for microscopy.

### 4.14. Statistical Analyses

Experimental replicates and numbers of mice are stated in the respective figure legends. Data are presented as mean ± standard deviation (SD) and analyzed using GraphPad Prism v.10 (GraphPad Software, San Diego, CA, USA). One-way ANOVA in conjunction with Dunnett tests was used for statistical analyses. *p* < 0.05 was considered statistically significant.

## 5. Conclusions

In summary, BBR was identified as the most active alkaloid in our screening system and alleviated *C. rodentium*-induced colitis with reduced epithelial GSDME cleavage, restrained HMGB1/ERK/JNK inflammatory signaling, and improved barrier-related readouts. Because this effect occurred without significantly reducing bacterial burden, BBR appears to act mainly by limiting bacteria-associated epithelial pyroptotic injury. These findings support BBR as a promising candidate for the treatment of colitis driven by epithelial pyroptosis.

## Data Availability

The datasets used during the current study are available from the corresponding author on reasonable request.

## Supplementary Materials

The following supporting information can be downloaded at https://www.mdpi.com/article/10.3390/ijms27156820/s1.

## Abbreviations

The following abbreviations are used in this manuscript:

BBR	Berberine
CHX	Cycloheximide
ERK	Extracellular signal-regulated kinase
GSDMD	Gasdermin D
GSDME	Gasdermin E
HMGB1	High mobility group box 1
IBD	Inflammatory bowel disease
IECs	Intestinal epithelial cells
JNK	C-Jun N-terminal kinase
NLRP3	NOD-like receptor pyrin domain-containing 3
TNF-a	Tumor necrosis factor-alpha
ZO-1	Zonula occludens-1

## References

[1] Jairath V., Narula N., Ungaro R.C., Romo Bautista I., Adsul S. (2025). Novel outcomes in inflammatory bowel disease. J. Crohns Colitis.

[2] de Souza H.S.P., Fiocchi C., Iliopoulos D. (2017). The IBD interactome: An integrated view of aetiology, pathogenesis and therapy. Nat. Rev. Gastroenterol. Hepatol..

[3] Kappelman M.D., Farkas D.K., Long M.D., Erichsen R., Sandler R.S., Sørensen H.T., Baron J.A. (2014). Risk of cancer in patients with inflammatory bowel diseases: A nationwide population-based cohort study with 30 years of follow-up evaluation. Clin. Gastroenterol. Hepatol..

[4] Wan J., Zhou J., Wang Z., Liu D., Zhang H., Xie S., Wu K. (2025). Epidemiology, pathogenesis, diagnosis, and treatment of inflammatory bowel disease: Insights from the past two years. Chin. Med. J..

[5] Patankar J.V., Becker C. (2020). Cell death in the gut epithelium and implications for chronic inflammation. Nat. Rev. Gastroenterol. Hepatol..

[6] Gustafsson J.K., Johansson M.E.V. (2022). The role of goblet cells and mucus in intestinal homeostasis. Nat. Rev. Gastroenterol. Hepatol..

[7] Lin Q., Zhang S., Zhang J., Jin Y., Chen T., Lin R., Lv J., Xu W., Wu T., Tian S. (2025). Colonic epithelial-derived FGF1 drives intestinal stem cell commitment toward goblet cells to suppress inflammatory bowel disease. Nat. Commun..

[8] Peterson L.W., Artis D. (2014). Intestinal epithelial cells: Regulators of barrier function and immune homeostasis. Nat. Rev. Immunol..

[9] Broz P. (2025). Pyroptosis: Molecular mechanisms and roles in disease. Cell Res..

[10] Jena K.K., Mambu J., Boehmer D., Sposito B., Millet V., de Sousa Casal J., Muendlein H.I., Spreafico R., Fenouil R., Spinelli L. (2024). Type III interferons induce pyroptosis in gut epithelial cells and impair mucosal repair. Cell.

[11] Wang Y., Gao W., Shi X., Ding J., Liu W., He H., Wang K., Shao F. (2017). Chemotherapy drugs induce pyroptosis through caspase-3 cleavage of a gasdermin. Nature.

[12] Li Y., Yuan Y., Huang Z.X., Chen H., Lan R., Wang Z., Lai K., Chen H., Chen Z., Zou Z. (2021). GSDME-mediated pyroptosis promotes inflammation and fibrosis in obstructive nephropathy. Cell Death Differ..

[13] Xue R., Li M., Zhang G., Zhang W., Han L., Bo T., Zhong H., Yao D., Deng Y., Chen S. (2024). GSDME-mediated pyroptosis contributes to chemotherapy-induced platelet hyperactivity and thrombotic potential. Blood.

[14] Cabral J.E., Wu A., Zhou H., Pham M.A., Lin S., McNulty R. (2025). Targeting the NLRP3 inflammasome for inflammatory disease therapy. Trends Pharmacol. Sci..

[15] Tan G., Huang C., Chen J., Zhi F. (2020). HMGB1 released from GSDME-mediated pyroptotic epithelial cells participates in the tumorigenesis of colitis-associated colorectal cancer through the ERK1/2 pathway. J. Hematol. Oncol..

[16] Wu Y.Z., Xie Y., Chen L., Ning L., Hu X.Q., Xu X.P. (2025). Gasdermin E-mediated intestinal epithelial pyroptosis promotes chemically induced colitis in mice. Gastroenterol. Rep..

[17] Xiao J., Sun K., Wang C., Abu-Amer Y., Mbalaviele G. (2022). Compound loss of GSDMD and GSDME function is necessary to achieve maximal therapeutic effect in colitis. J. Transl. Autoimmun..

[18] Zhou Q., Liu L., Lin X., Shen B., Huang W., Zhang Y., Li Y., Yang Y., Liu H., Zhang W. (2025). Nicotinamide riboside attenuates radiation-induced intestinal injury by suppressing gasdermin E-mediated pyroptosis in intestinal epithelial cells. J. Transl. Med..

[19] Li W., Chen D., Zhu Y., Ye Q., Hua Y., Jiang P., Xiang Y., Xu Y., Pan Y., Yang H. (2024). Alleviating Pyroptosis of Intestinal Epithelial Cells to Restore Mucosal Integrity in Ulcerative Colitis by Targeting Delivery of 4-Octyl-Itaconate. ACS Nano.

[20] Yang W., Wang Y., Wang T., Li C., Shi L., Zhang P., Yin Y., Tao K., Li R. (2023). Protective effects of IRG1/itaconate on acute colitis through the inhibition of gasdermins-mediated pyroptosis and inflammation response. Genes Dis..

[21] Xie Q., Li H., Ma R., Ren M., Li Y., Li J., Chen H., Chen Z., Gong D., Wang J. (2022). Effect of *Coptis chinensis* franch and *Magnolia officinalis* on intestinal flora and intestinal barrier in a TNBS-induced ulcerative colitis rats model. Phytomedicine.

[22] Zhou R., Huang Y., Tian C., Yang Y., Zhang Z., He K. (2023). *Coptis chinensis* and Berberine Ameliorate Chronic Ulcerative Colitis: An Integrated Microbiome-Metabolomics Study. Am. J. Chin. Med..

[23] Yang Y., Ren R., Chen Q., Zhang Q., Wu J., Yin D. (2022). *Coptis chinensis* polysaccharides dynamically influence the paracellular absorption pathway in the small intestine by modulating the intestinal mucosal immunity microenvironment. Phytomedicine.

[24] Li N., Shang X., Shi L., Li Y., Mao T., Wang Q., Li J., Peng G. (2025). Effects of three Chinese herbal therapies on gut microbiota and short-chain fatty acid metabolism in patients with mild, moderate, and severe ulcerative colitis: Multi-center, randomized, controlled trials. Int. Immunopharmacol..

[25] Zhang J.X., Hu Y.X., Liu Y., Chen Z.Z., Zheng J.T., Qu X.T., Zhang Y., Tang W.Y., Huang S.C., Liu C.S. (2024). Xianglian pill alleviates ulcerative colitis by inhibiting M1 macrophage polarization via modulation of energy metabolite itaconate. Phytomedicine.

[26] Dai Y., Lu Q., Li P., Zhu J., Jiang J., Zhao T., Hu Y., Ding K., Zhao M. (2023). Xianglian Pill attenuates ulcerative colitis through TLR4/MyD88/NF-κB signaling pathway. J. Ethnopharmacol..

[27] Li Q., Cui Y., Xu B., Wang Y., Lv F., Li Z., Li H., Chen X., Peng X., Chen Y. (2021). Main active components of Jiawei Gegen Qinlian decoction protects against ulcerative colitis under different dietary environments in a gut microbiota-dependent manner. Pharmacol. Res..

[28] Li H., Feng C., Fan C., Yang Y., Yang X., Lu H., Lu Q., Zhu F., Xiang C., Zhang Z. (2020). Intervention of oncostatin M-driven mucosal inflammation by berberine exerts therapeutic property in chronic ulcerative colitis. Cell Death Dis..

[29] Jing W., Dong S., Luo X., Liu J., Wei B., Du W., Yang L., Luo H., Wang Y., Wang S. (2021). Berberine improves colitis by triggering AhR activation by microbial tryptophan catabolites. Pharmacol. Res..

[30] Yan S., Chang J., Hao X., Liu J., Tan X., Geng Z., Wang Z. (2022). Berberine regulates short-chain fatty acid metabolism and alleviates the colitis-associated colorectal tumorigenesis through remodeling intestinal flora. Phytomedicine.

[31] Dong Y., Fan H., Zhang Z., Jiang F., Li M., Zhou H., Guo W., Zhang Z., Kang Z., Gui Y. (2022). Berberine ameliorates DSS-induced intestinal mucosal barrier dysfunction through microbiota-dependence and Wnt/β-catenin pathway. Int. J. Biol. Sci..

[32] Zhang J.L., Zhang M.N., Wang H.G., Yang X.Z., Yu C.G. (2022). Jatrorrhizine alleviates ulcerative colitis via regulating gut microbiota and NOS2 expression. Gut Pathog..

[33] Tan B., Zhang J., Kang A., Zhang L., Fang D., Wu H., Han T., Qiu R., Li H., Sun D. (2025). Coptisine activates aryl hydrocarbon receptor to regulate colonic epithelial homeostasis in DSS induced ulcerative colitis and TNF-α challenged intestinal organoids. Phytomedicine.

[34] Chen M., Wang Y., Chen L., Chen M., Li X., Wang G. (2025). Epiberberine ameliorates ulcerative colitis by regulating bile acids hepatoenteral circulation through intestinal FXR. Phytomedicine.

[35] Ji W., Zhang Y., Qian X., Hu C., Huo Y. (2024). Palmatine alleviates inflammation and modulates ferroptosis against dextran sulfate sodium (DSS)-induced ulcerative colitis. Int. Immunopharmacol..

[36] Huang C.Y., Chiang S.F., Chen W.T., Ke T.W., Chen T.W., You Y.S., Lin C.Y., Chao K.S.C., Huang C.Y. (2018). HMGB1 promotes ERK-mediated mitochondrial Drp1 phosphorylation for chemoresistance through RAGE in colorectal cancer. Cell Death Dis..

[37] Wu H., Chen Z., Xie J., Kang L.N., Wang L., Xu B. (2016). High Mobility Group Box-1: A Missing Link between Diabetes and Its Complications. Mediat. Inflamm..

[38] Mullineaux-Sanders C., Sanchez-Garrido J., Hopkins E.G.D., Shenoy A.R., Barry R., Frankel G. (2019). *Citrobacter rodentium*-host-microbiota interactions: Immunity, bioenergetics and metabolism. Nat. Rev. Microbiol..

[39] Yao D., Dai W., Dong M., Dai C., Wu S. (2021). MUC2 and related bacterial factors: Therapeutic targets for ulcerative colitis. eBioMedicine.

[40] Liu Y., Yu Z., Zhu L., Ma S., Luo Y., Liang H., Liu Q., Chen J., Guli S., Chen X. (2023). Orchestration of MUC2—The key regulatory target of gut barrier and homeostasis: A review. Int. J. Biol. Macromol..

[41] Miao G., Zhang D., Sui Z., Leng J., Deng Y., Gu X., Cao H. (2025). Berberine-taxifolin co-administration attenuates inflammatory response and intestinal barrier injury via nf-κB/NLRP3 suppression in colitis. Front. Immunol..

[42] Li H., Fan C., Lu H., Feng C., He P., Yang X., Xiang C., Zuo J., Tang W. (2020). Protective role of berberine on ulcerative colitis through modulating enteric glial cells-intestinal epithelial cells-immune cells interactions. Acta Pharm. Sin. B.

[43] Li J., Dan W., Zhang C., Liu N., Wang Y., Liu J., Zhang S. (2024). Exploration of Berberine Against Ulcerative Colitis via TLR4/NF-κB/HIF-1α Pathway by Bioinformatics and Experimental Validation. Drug Des. Dev. Ther..

[44] Xu L., Zhang Y., Xue X., Liu J., Li Z.S., Yang G.Y., Song Y., Pan Y., Ma Y., Hu S. (2020). A Phase I Trial of Berberine in Chinese with Ulcerative Colitis. Cancer Prev. Res..

[45] Li J., Zhang C., Xu Y., Yang L. (2024). Efficacy and safety of berberine plus 5-ASA for ulcerative colitis: A systematic review and meta-analysis. PLoS ONE.

[46] Tan G., Huang C., Chen J., Chen B., Zhi F. (2021). Gasdermin-E-mediated pyroptosis participates in the pathogenesis of Crohn’s disease by promoting intestinal inflammation. Cell Rep..

[47] Liu K., Song M., Huang X., Shi Y., Li S., Zhu F., Ben T., Lin X., Chen B., Xu B. (2024). Western diet induces GSDME-mediated epithelial pyroptosis through the DCA-S1PR2 pathway to disrupt the intestinal epithelial barrier. Sci. Bull..

[48] Takahara M., Takaki A., Hiraoka S., Adachi T., Shimomura Y., Matsushita H., Nguyen T.T.T., Koike K., Ikeda A., Takashima S. (2019). Berberine improved experimental chronic colitis by regulating interferon-γ- and IL-17A-producing lamina propria CD4(+) T cells through AMPK activation. Sci. Rep..

[49] Wang Z., Zhong Y., Xin M., Zhang J., Dong X., Zhang W., Lu X., Li L., Tu Y., Zhang L. (2024). Swiprosin-1 participates in the berberine-regulated AMPK/MLCK pathway to attenuate colitis-induced tight junction damage. Phytomedicine.

[50] Ren G., Ding Y.W., Wang L.L., Jiang J.D. (2023). Berberine stimulates lysosomal AMPK independent of PEN2 and maintains cellular AMPK activity through inhibiting the dephosphorylation regulator UHRF1. Front. Pharmacol..

[51] Ai Y.L., Wang W.J., Liu F.J., Fang W., Chen H.Z., Wu L.Z., Hong X., Zhu Y., Zhang C.X., Liu L.Y. (2023). Mannose antagonizes GSDME-mediated pyroptosis through AMPK activated by metabolite GlcNAc-6P. Cell Res..

[52] Zhang M.Q., Jia X., Cheng C.Q., Wang Y.X., Li Y.Y., Kong L.D., Li Q.Q., Xie F., Yu Y.L., He Y.T. (2023). Capsaicin functions as a selective degrader of STAT3 to enhance host resistance to viral infection. Acta Pharmacol. Sin..

[53] Qiu M., Yi Q., Luo M., Duan S., Zhang Z., Wu X., Chen T., Ma C., Cui T., Zhang B. (2026). *Gynostemma pentaphyllum* polysaccharides alleviate ulcerative colitis in mice via the Nrf2/ROS/NLRP3 axis and modulation of the gut microbiota. J. Ethnopharmacol..

