# Supplementary material for: Berberine Alleviates Citrobacter rodentium-Induced Colitis with Reduced Epithelial Gasdermin E-Mediated Pyroptosis
Source: Int J Mol Sci. 2026 Jul 29;27(15):6820. doi: 10.3390/ijms27156820 (PMC13466967; doi:10.3390/ijms27156820)
Supplement: Supplementary file 1 [file ijms-27-06820-s001.zip › ijms-4399258-supplementary.pdf]

Supplemental file

Titel:

Berberine alleviates *Citrobacter rodentium*-induced colitis with reduced epithelial gasdermin E-mediated pyroptosis

Supplementary Table s1. Primers for qPCR.

Genes	Species	Primer sequence (5′-3′)
Actb	Mouse	F-GTGACGTTGACATCCGTAAAGA<br>R-GCCGGACTCATCGTACTCC
Il1b	Mouse	F-CTGTGACTCATGGGATGATGATG<br>R-CGGAGCCTGTAGTGCAGTTG
Tnf	Mouse	F-CAGGCGGTGCCTATGTCTC<br>R-CGATCACCCCGAAGTTCAGTAG
Cxcl1	Mouse	F-ACTGCACCCAAACCGAAGTC<br>R-TGGGGACACCTTTTAGCATCTT
Cxcl2	Mouse	F-CCAACCACCAGGCTACAGG<br>R-GCGTCACACTCAAGCTCTG
Cxcl3	Mouse	F-AAGTTTGCCTCAACCCCCAA<br>R-GGACTTGCCGCTCTTCAGTA
Cxcl5	Mouse	F-TGCGTTGTGTTTGCTTAACCG<br>R-CTTCCACCGTAGGGCACTG
Muc2	Mouse	F-CTGACCAAGAGCGAACACAA<br>R-CATGACTGGAAGCAACTGGA<br>R-CTGAAGTTTTCTCCATAAGGGCT
Tjp1	Mouse	F-<br>CGCCTTCATACAATAAAGCAAATCATAG<br>R-ACTGCGCATAATTAAGACGATCAAC
Cldn1	Mouse	F-CCCCCATCAATGCCAGGTA<br>R-GGACAGGAGCAGGAAAGTAGGAC
ACTB	Human	F-CATGTACGTTGCTATCCAGGC<br>R-CTCCTTAATGTCACGCACGAT

IL1B	Human	F-AGCTACGAATCTCCGACCAC<br>R-CGTTATCCCATGTGTCGAAGAA
TNF	Human	F-CCTCTCTCTAATCAGCCCTCTG<br>R-GAGGACCTGGGAGTAGATGAG
CXCL5	Human	F-AGCTGCGTTGCGTTTGTTTAC<br>R-TGGCGAACACTTGCAGATTAC

Note: F, Forward primer；R, Reversed primer.

Supplementary Table s2. Histopathological scoring criteria for H&E staining score

	Criteria for Histological Damage Assessment	Criteria for Inflammatory Cell Infiltration Evaluation
**0**	Essentially absent mucosal injury	Sparse inflammatory cell accumulation in the mucosal lamina propria
**1**	Discrete areas of superficial mucosal epithelial disruption	Increased infiltration of inflammatory cells within the mucosal lamina propria
**2**	Marked erosion of the superficial mucosal layer	Substantial inflammatory cell infiltration extending into the submucosal compartment
**3**	Extensive full-thickness mucosal breakdown	Widespread inflammatory cell migration throughout the entire tissue architecture

Supplementary Figure

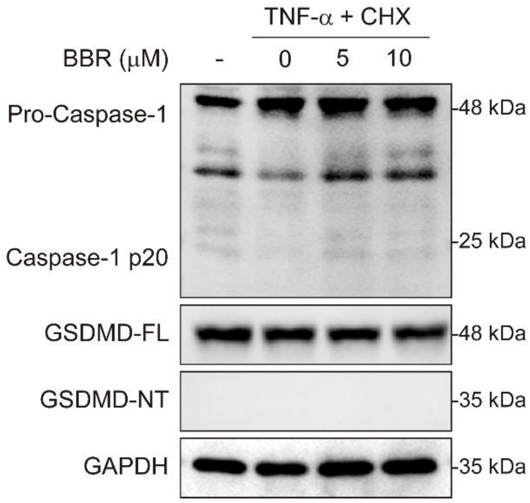

Supplementary Figure s1. The effect of Berberine on Caspase-1 and GSDMD activation. HCT116 cells were pretreated with BBR for 4 h and subsequently co-incubated with TNF-⊚/CHX for 16 h. Protein expression levels of Caspase-1 and GSDMD were determined by Western blotting.

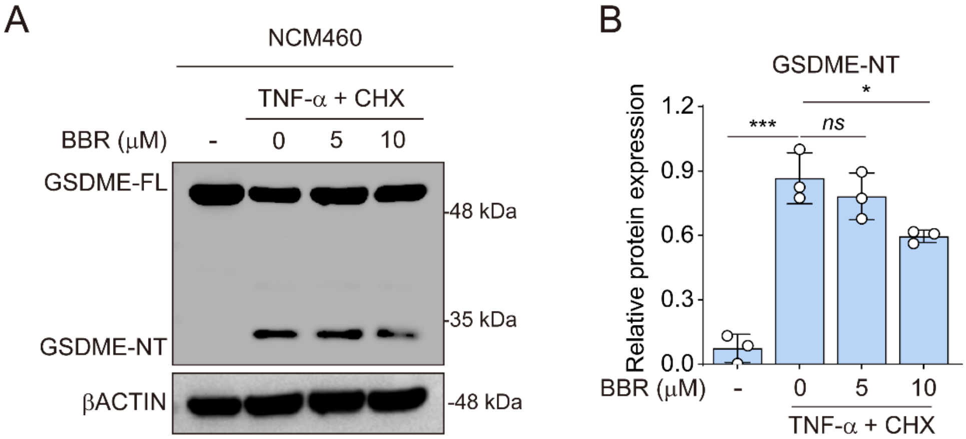

Supplementary Figure s2. The effect of Berberine on GSDME activation in NCM460 cells. (A, B) NCM460 cells were pretreated with BBR for 4 h and subsequently co-incubated with TNF-◎/CHX for 9 h. Protein expression level of GSDME was determined by Western blotting. The relative grayscale value of the cleaved GSDME-NT protein band was measured with ImageJ software (n = 3). Data are presented as mean ± SD. ns = not significant; *P < 0.05, and ***P < 0.001 versus model group.

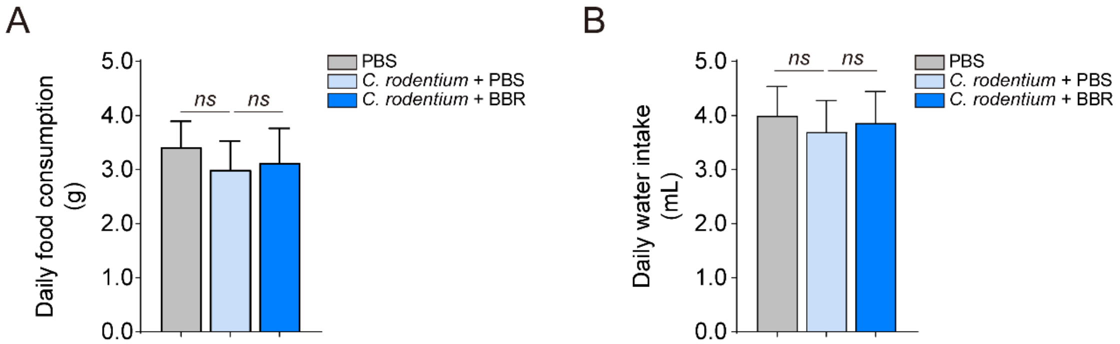

Supplementary Figure s3. Effect of Berberine on water and food intake in infected mice. Mice were treated with BBR for 1 day prior to the infection with *C. rodentium* for 7 days (n = 5). (A-B) Daily food consumption intake and daily water intake of *C. rodentium* infection mice with or without BBR treated. Data are mean ± SD. *ns* = not significant.
